# Application of Antisense Conjugates for the Treatment of Myotonic Dystrophy Type 1

**DOI:** 10.3390/ijms24032697

**Published:** 2023-01-31

**Authors:** Jessica Stoodley, Francisco Vallejo-Bedia, David Seone-Miraz, Manuel Debasa-Mouce, Matthew J. A. Wood, Miguel A. Varela

**Affiliations:** 1 Department of Paediatrics, Institute of Developmental and Regenerative Medicine (IDRM), University of Oxford, Roosevelt Dr, Oxford OX3 7TY, UK; 2 MDUK Oxford Neuromuscular Centre, Oxford OX3 7TY, UK

**Keywords:** antibody, bridged nucleic acids, cell-penetrating peptide, lipids, muscle, myotonic dystrophy, oligonucleotides, splicing

## Abstract

Myotonic dystrophy type 1 (DM1) is one of the most common muscular dystrophies and can be potentially treated with antisense therapy decreasing mutant DMPK, targeting miRNAs or their binding sites or via a blocking mechanism for MBNL1 displacement from the repeats. Unconjugated antisense molecules are able to correct the disease phenotype in mouse models, but they show poor muscle penetration upon systemic delivery in DM1 patients. In order to overcome this challenge, research has focused on the improvement of the therapeutic window and biodistribution of antisense therapy using bioconjugation to lipids, cell penetrating peptides or antibodies. Antisense conjugates are able to induce the long-lasting correction of DM1 pathology at both molecular and functional levels and also efficiently penetrate hard-to-reach tissues such as cardiac muscle. Delivery to the CNS at clinically relevant levels remains challenging and the use of alternative administration routes may be necessary to ameliorate some of the symptoms experienced by DM1 patients. With several antisense therapies currently in clinical trials, the outlook for achieving a clinically approved treatment for patients has never looked more promising.

## 1. Introduction

Myotonic dystrophy type 1 (DM1) is the most common form of muscular dystrophy found in adults with a prevalence of 1 in 8000 people [1,2] although it has been reported that the genetic cause of DM1 is 3–4 times more common and mild forms of the disease are underdiagnosed [3]. In severe forms of the disease, individuals can be affected from birth [4]. The disease is characterised as a progressive, autosomal dominant, genetic disorder that affects multiple organs and systems. The clinical characteristics of DM1 vary greatly between patients but commonly include severe muscle weakness and myotonia, cardiac conduction defects, cataracts, insulin resistance and, more typically in younger patients, cognitive and neurological dysfunction. DM1 is caused by an expansion of CTG tandem repeats in the 3′-untranslated region of the myotonic dystrophy protein kinase (*DMPK*) gene which, upon transcription, leads to an RNA gain of function mutation [5,6]. Unaffected individuals have CTG repeat lengths of 5–37, while CTG repeats of over 50 lead to the manifestation of disease symptoms [7]. The severity of the disease is proportional to the number of CTG repeats, with increasing repeat lengths typically giving rise to an earlier onset of disease symptoms, increased symptom severity and increased somatic instability within tissues [7,8].

The muscleblind-like protein (MBNL) family has a main role in the regulation of mRNA metabolism, specifically post-transcriptional alternative splicing [9]. The family comprises three proteins with different lifetime expression patterns: MBNL1 and MBNL2 are expressed postnatally, while MBNL3 is expressed specifically during the embryonic phase and in regenerating adult tissues (e.g., liver and bone marrow) [10]. CUG repeat-expanded *DMPK* transcripts are retained in the nucleus as ribonucleoprotein foci and are able to sequester MBNL proteins, leading to a loss of protein function [11,12,13,14]. This alters the proteins’ splicing regulatory activity, generating predominantly foetal patterning of splicing and resulting in the identified alternative splicing of over 30 human genes [15]. Some of the most studied include CLCN1 [16], DMD [17], BIN1 [18], SCN5A [19] and INSR [20] and their missplicing can be directly correlated with classic DM1 symptoms such as muscle weakness [18], cardiac conduction defects [19] and insulin resistance [20]. Furthermore, MBNL1 is a negative regulator of the CUGBP Elav-like family member 1 (CELF1) protein, which is hyper-phosphorylated and upregulated in DM1, giving rise to further splicing misregulation [21,22,23]. Additionally, studies have shown that RNA localisation and trafficking, poly-adenylation, protein translation and micro-RNA processing are also affected by the altered behaviour of MBNL and CELF proteins in DM1 pathology [15,24,25].

There are a number of DM1 mouse models that have been used for proof-of-concept studies and the preclinical development of therapeutic approaches. These mouse models recapitulate DM1 pathology partially and they can be considered complementary. For example, the HSA-LR is a transgenic mouse expressing 250 untranslated CUGs in the human skeletal actin (HSA) that display missplicing and myotonia, but the transgene is only expressed in skeletal muscle, not in cardiac muscle or the CNS [26]. Other transgenic mice with tetracycline-inducible heart-specific expression of human mutant *DMPK* mRNA with 960 repeats have been used to study the cardiac pathogenesis in DM1 [27], whereas the mouse model most commonly used to study DM1 pathology in the CNS is the DMSXL mouse, with more than 1000 CTGs in the human DM1 locus (45 Kb), muscle defects and high mortality, although with mild missplicing [28]. All these mouse models have been useful in the field, but there is a need to develop large animal models that recapitulate the delivery bottleneck of new therapies in human patients.

There is currently no curative treatment for DM1, with the only treatments available to patients being aimed at managing the disease symptoms. One key reason for this is the multi-systemic nature of the disease, which requires the targeting of treatments into the cell nuclei of a range of tissues. Critically affected tissues include skeletal muscle and heart, the membranes of which are challenging to traverse, and the central nervous system (CNS), a tissue protected by the blood–brain barrier (BBB). However, advancements in gene technologies and the treatment of several other neuromuscular diseases have led to a number of therapeutic approaches being explored for DM1. These therapeutic approaches can be grouped into three categories: antisense therapy, gene therapy and small molecule drugs [29]. We and many others in this field believe that future therapeutic interventions could involve a combination of modalities, for example, an initial treatment with gene therapy followed by the repetitive administration of antisense therapy or a small molecule. Targeting RNA with antisense therapy does not elicit immune responses like the AAVs regularly used in gene therapy and provides the precision needed to avoid the off-target effects of many small molecules. This review will focus on the use and development of antisense therapy for the treatment of DM1, discussing the hurdles and current progress towards future clinical applications.

## 2. ASO Therapeutics

Antisense oligonucleotides (ASOs) are small (~14–30 nucleotides), single-stranded synthetic nucleic acid polymers which can be employed to modulate gene expression. ASOs can be divided into two classes, based on their mechanism of action: RNase H1-dependent ASOs or steric blocking ASOs. RNase H1-dependent ASOs form a duplex with the target RNA sequence which is recognised by RNase H1. The recruitment of RNase H1 to the duplex results in the cleavage of the target RNA and therefore a downregulation in gene expression [30,31]. Typically, ASOs acting by this mechanism are termed “*gapmers*” as they are comprised of a central stretch of approximately 10 nucleotides complementary to the target RNA sequence, flanked on each side by 2–5 chemically modified nucleotides [32]. The chemical modifications render the flanking regions unrecognisable to RNAse H1 but are beneficial to the overall compound due to their ability to increase the binding affinity to target RNA. Steric blocking ASOs, on the other hand, lack RNase H1 competence due to chemical modifications across the ASO and so fail to induce target degradation. Instead, they are designed to bind to target transcripts with a high affinity and interfere with RNA–RNA or RNA–protein interactions. Their clinical applications are assorted but often include modulation of alternative splicing, via exon inclusion or exclusion [33,34]. RNA gain-of-function diseases can be also treated with steric block ASOs designed to corrupt the toxic RNA function [35,36,37] while approaches to restore the translational reading frame are being employed to loss-of-function diseases [38].

Oligonucleotides have promising clinical potential due to their high target specificity, but poor biodistribution outside liver and kidney when injected systemically and they are susceptible to degradation by endonucleases and exonucleases [39]. Consequently, in order to enhance delivery and potency, a number of chemical modifications have been deployed over the years, to improve ASO pharmacological characteristics [40]. Phosphorothioation of the ASO backbone (PS), whereby a non-bridging oxygen atom is replaced with a sulphur, has been shown to improve ASO resistance to nuclease activity and increase serum half-life [41,42,43]. Meanwhile, modifications at the 2′ position of the ribose sugar have led to the development of a number of ASOs with improved safety, improved pharmokinetics and reduced immunostimulatory activity [44,45]. Bridged nucleic acid chemistries introduce a chemical bridge between the 2′ and 4′ position on the ribose ring of the ASO [46,47,48,49,50]. This constrains the pucker of the ribose sugar to the 3′ endo conformation, increasing the ASO affinity for its RNA target and enhancing stability against nucleases. Additionally, ASOs with charge-neutral backbones have been developed, including phosphorodiamidate morpholino oligonucleotide (PMOs) where the nucleotide backbone is replaced with neutral 6-membered morpholino rings [51] and peptide nucleic acids (PNAs) [52] where the ASO backbone consists of a pseudopeptide analogue. In both cases, the overall neutrality of the ASO charge increases its serum stability while facilitating the potential for bioconjugation to other chemical moieties that may aid compound delivery to target tissues [53].

## 3. Antisense Therapeutics for DM1

The majority of ASO therapies in development for DM1 have focused on muscle as the primary tissue target, although CNS is also an important target tissue, especially for the congenital form of DM1. Both RNAse H1-dependent and steric block-modified ASO approaches have been used as strategies in the preclinical development of therapies for DM1. RNAse H1-dependent DM1 gapmers are designed to bind to repeat expanded *DMPK* RNA, causing its degradation and a reduction in sequestered MBNL1 and resultant missplicing events [54,55,56,57]. Meanwhile, steric block ASOs bind with a high affinity to *DMPK* CUG RNA repeats, thereby preventing the binding of MBNL1 and eliciting correction of splicing [35,36,58]. For example, CAG25, a naked 25-base pair PMO that sterically blocks MBNL1 sequestration to CUG-expanded *DMPK* RNA, was shown to restore splicing abnormalities, reverse myotonia and reduce the number RNA foci in a DM1 mouse model upon intramuscular injection. The compound did not reduce endogenous levels of expanded CUG transcripts in wild-type (WT) mice, demonstrating a proof-of-concept for steric block DM1 ASOs selecting disease *DMPK* transcripts over WT. However, systemic injection failed to correct key missplicing events [59]. Similarly, several ASO gapmers have been successfully used in cellular and animal DM1 models to treat the molecular phenotype of the disease [54,55,56,57].

### 3.1. Bridged Nucleic Acid Therapy

Locked nucleic acids (LNAs) are a form of bridged nucleic acid modification that can be introduced into ASOs to enhance stability and target-binding affinity. A single LNA subunit substitution into RNA or DNA oligomer typically increases the Tm by 2–8 °C, greatly enhancing thermal stability [60]. Nucleic acids containing LNA subunits have been used as *mixmers* which contain several LNA subunits in random positions within an ASO; or as *gapmers* with several LNA subunits on both sides of the ASO [48,49]. Alternatively, LNA technology can also be used to synthesise all-LNA oligomers, capable of stably binding target DNA or RNA molecules without causing their endonucleolytic cleavage by RNase H [61,62]. For example, PO-LNA-CAG-10, an all-LNA ASO complementary to CUG repeats, was able to specifically correct MBNL-sensitive alternative splicing abnormalities and reduce the number and size of CUG repeat-expanded foci in vitro, although the compound was unable to enter cells without transfection agents [62]. Subsequent intramuscular injections of PO-LNA-CAG-10 in DM1 mice (HSA^LR^) showed the promising correction of the alternative splicing patterns in skeletal muscles to near wild type levels, 48 h after treatment. More recently, a combination of 2′OMe monomers with LNA nucleotides in every third position was able to improve *Mbnl1* exon 7 missplicing in primary HSA^LR^ myoblasts by directly competing with Mbnl1 for CUG binding, in order to restore protein function. Intramuscular delivery to the tibialis anterior muscle in HSA^LR^ mice resulted in the amelioration of the alternative splicing of Mbnl1-dependant exons. However, systemic subcutaneous injection of the LNA/2′-O-Methyl ASOs within the same mouse model, while exhibiting low toxicity, failed to correct missplicing of *Serca2* and reduce the number of CUG-expanded foci within the skeletal muscles. It was overall concluded that the skeletal muscle uptake of their LNA/2′-O-Methyl ASOs was insufficient for intracellular efficacy without bioconjugation to a tissue-targeting moiety.

Other bridged nucleic acid chemistries such as cEt (2′,4′-constrained ethyl) have been more successful at normalising DM1 muscle phenotype after systemic administration in DM1 mice [56]. However, clinical study data suggest that the use of a systemically delivered naked ASO alone is insufficient for achieving the desired potency or efficacy in skeletal muscles in patients, even when chemically modified. The first ASO compound to be tested for DM1 in clinical trials was a naked cEt-modified gapmer developed by IONIS. The compound had previously shown high efficacy in reversing the DM1 skeletal muscle phenotype and low toxicity in preclinical animal models [63]. However, the clinical trial (NCT02312011) was discontinued due to a failure to achieve skeletal muscle concentrations required for functional and biological efficacy [64]. Therefore, further enhancements such as bioconjugation or alternative ASO strategies and delivery methods are being developed in order to improve the stability and biodistribution of ASOs for the treatment of DM1.

### 3.2. Bioconjugation: Lipid Conjugates

**Figure 1:**
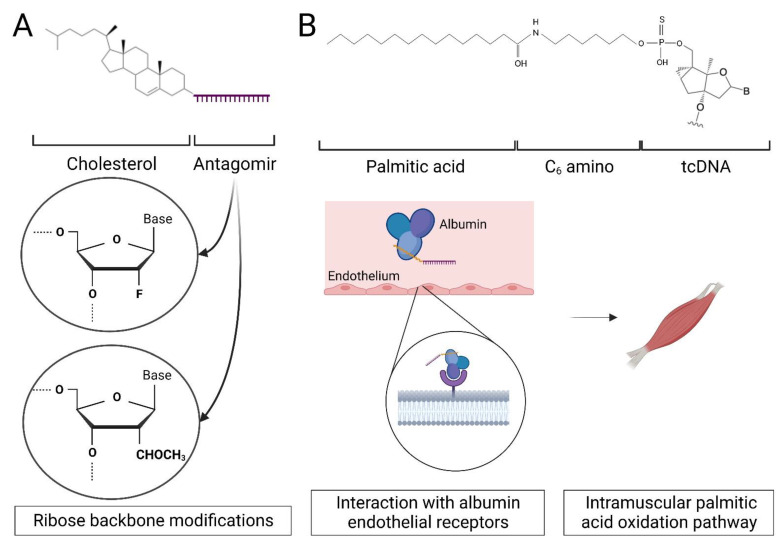
Antisense molecules can be conjugated to lipids to improve their pharmacokinetic properties. (**A**) Single-stranded RNA analogues complementary to miRNAs (AntagomiR) conjugated to cholesterol are delivered to non-CNS tissues at clinically relevant concentrations. Crossing the BBB into the CNS can be improved by conjugating the lipid to a DNA/RNA heteroduplex oligonucleotide [71]. (**B**) Conjugation of tricyclo-DNA (tcDNA) [72] ASOs with fatty acids such as palmitic acid has also been shown to improve ASO distribution by binding to serum albumin and indirectly with endothelial surface receptors facilitating transport across the continuous capillary endothelium in muscle.

Bioconjugation involves the direct covalent linkage of various chemical moieties to ASOs and is now widely used to enhance intracellular uptake, tissue specificity and reduce renal clearance from systemic circulation [40]. ASO conjugation with hydrophobic lipid moieties improves distribution to skeletal and cardiac muscle in both mice and non-human primates, enhancing association with lipoproteins such as HDL and albumin [65] (Figure 1). Albumin is the most abundant plasma protein in human blood, with circulating levels of 35–50 g/L [66]. As skeletal and cardiac muscle cells rely on the oxidation of long-chain fatty acids for contractile work, fatty acids are transported through blood to these cells and cross the tissue endothelium via albumin interactions with endothelial surface receptors [67,68]. Conjugation of PS ASOs with fatty acids, such as palmitic acid, improves compound affinity for serum albumin decreasing renal clearance and therefore potency in muscle tissues [69]. Moreover, lipid conjugation enhances the endosomal release of ASOs in cells [70].

In the context of DM1, the group of Ruben Artero (University of Valencia) developed DM1 therapeutic antisense compounds conjugated to lipids that silence microRNAs (miRNAs) associated with the disease pathology [73]. These compounds, called antagomiRs or anti-miRs [74], are steric blocking ASOs that are complementary to miRNAs and also chemically modified at different positions in the backbone and on the sugar molecule [75]. AntagomiR-23b silences miRNA-23b-3p leading to an increase in MBNL protein levels which can ameliorate DM1 pathology [73]. The effect of antagomiR-23b was studied via subcutaneous and intravenous administrations in the DM1 HSA^LR^ mice and demonstrated an improvement in skeletal muscle phenotype after single injections at 12.5 mg/kg [76]. Further analysis showed MBLN1 splicing correction and increased expression, with restored normal MBNL1/2 distribution at the nucleus and cytoplasm level in muscle fibres. The presence of RNA *foci* remained unchanged [73]. The silencing of has-miR-218-5p, an endogenous MBNL1/2 repressor in human myotubes, overexpressed in DM1 animal disease models and patient muscle biopsies also led to MBNL1/2 upregulation in myotubes [73]. The systemic administration of antagomiR-218 to HSA^LR^ mice induced similar improvements in molecular and physical phenotypes as observed for antagomiR-23b [77].

### 3.3. Bioconjugation: Antibody Conjugates

**Figure 2:**
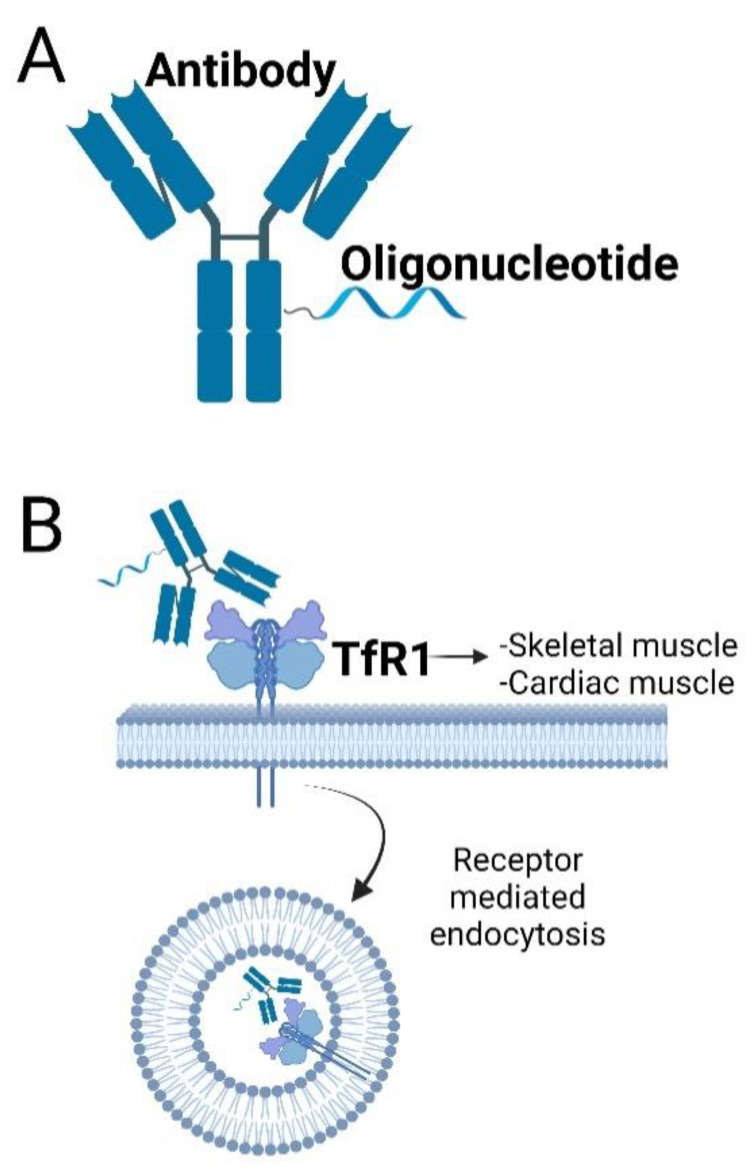
Antisense Oligonucleotides can be conjugated to antibodies targeting the Transferrin Receptor 1 (TfR1) and penetrate cells by receptor-mediated endocytosis. (**A**) Schematic design of an antibody–ASO conjugate. The ASO is conjugated directly to the antibody using covalent unions. (**B**) Antibodies targeting TfR1, a highly expressed cell-surface receptor of DM1 in target tissues such as skeletal and cardiac muscles, have been used to deliver antisense cargo. The AOC enters the cell by receptor-mediated endocytosis resulting on an enhanced ASO delivery. The affinity of TfR1antibodies can be fine-tuned to maximise the interaction with receptors in the BBB allowing for the compound to be delivered to the CNS (in mice) after systemic administration [85].

Antibody–drug conjugates have long been tested in oncology for targeting chemotherapeutic agents to specific tissues or cell types [78]. The conjugation of ASOs to antibodies shows promise for enhancing ASO delivery to DM1-affected tissues due the ability of antibodies to increase serum stability, membrane permeability and tissue selectivity [79]. The transferrin receptor, TfR1, is one of the most common cell-surface receptors to be exploited for antibody-facilitated delivery or oligonucleotides [80,81,82,83]. Antibody targeting of the TfR1 allows for the delivery of conjugated cargo into cells via receptor-mediated endocytosis and it is highly expressed in both skeletal and cardiac muscles (Figure 2). Two pharmaceutical companies (Avidity and Dyne Therapeutics) are exploiting this high TfR1 expression through the conjugation of DM1 small interfering RNA (siRNA) [84] targeting DMPK transcripts to TfR1 targeting antibodies or antibody fragments. The conjugation of TfR1-targeting antibodies or antibody fragments to siRNA-targeting DM1 repeat-expanded *DMPK* RNA allows for increased skeletal muscle penetration and therefore an overall enhancement in treatment efficacy. AOC1001 (Avidity), comprises of a monoclonal antibody against the TfR1 conjugated to a *DMPK* siRNA, and is currently being tested in a P1/2 clinical trial (MARINA: NCT05027269) for tolerability and safety at single and multiple doses [84]. In preclinical testing (data currently unpublished), the compound was reported to be successfully delivered to skeletal, cardiac and smooth muscles in both mouse and cynomolgus monkeys, producing a reduction in *DMPK* RNA expression and an amelioration of the disease phenotype in DM1 mice. Meanwhile, Dyne Therapeutics is recruiting patients for the clinical testing of Dyne-101, a *DMPK* ASO conjugated to an anti-TfR1 antibody fragment. Treatment of a DM1 mouse model with Dyne-101 caused a 40–50% and a 49% splicing correction in the skeletal muscle and heart biomarkers, respectively, while studies in cynomolgus monkeys did not reveal any toxicity after 13 weeks of treatment (ACHIEVE: NCT05481879).

### 3.4. Bioconjugation: Peptide Conjugates

**Figure 3:**
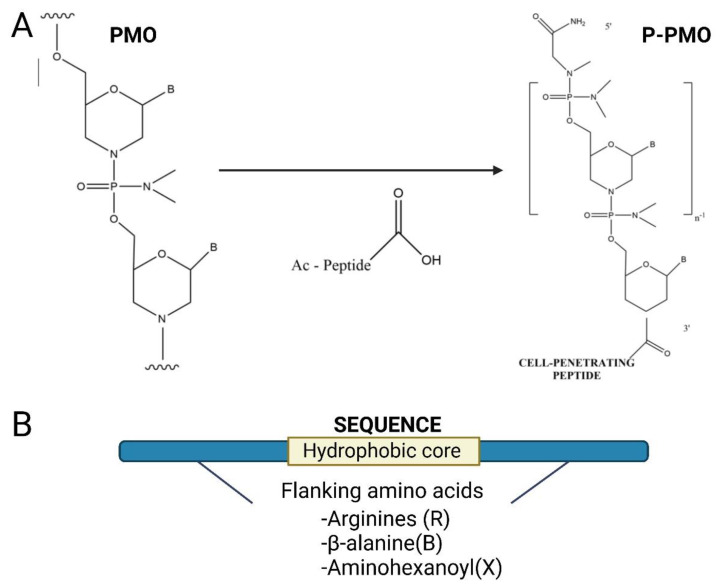
Antisense oligonucleotides can be conjugated to cell-penetrating peptides (CPPs) to increase cellular uptake. (**A**) CPPs can be directly conjugated to charge-neutral oligonucleotides, such as PMOs or PNAs using direct covalent unions [35]. (**B**) The peptide sequence is usually positively charged and rich in arginine. Here, a cell-penetrating peptide comprising a hydrophobic core and two flanking regions enriched with cationic amino acids such as arginine or B-alanine is shown.

Peptides can confer cell-penetrating, or tissue/cell targeting properties onto therapeutic ASOs. Cell-penetrating peptides (CPPs) can be directly conjugated to charge-neutral ASO chemistries, such as PMO and PNA, and this approach has been taken in several different splice-switching diseases [86,87,88,89,90,91]. The development of peptide PMOs (PPMOs) for the treatment of neuromuscular diseases in the groups of Matthew Wood (University of Oxford) and Mike Gait (MRC Laboratory of Molecular Biology, Cambridge) demonstrated the efficacy of this approach treating DM1, DMD and SMA with PMOs conjugated to different CPPs such as Pip6a [35,86,87] (Figure 3). Pip6a is a member of a series of PMO/PNA internalisation peptides (Pips) comprising arginines (R), β-alanine (B) and aminohexanoyl (X) amino acids flanking an internal core containing hydrophobic residues. The overall arginine-rich (RXRRBR)2XB-PMO structure is able to enhance ASO delivery to cardiac and skeletal muscle [89].

The conjugation of CPPs to PMOs was shown to improve the DM1 phenotype in HSA^LR^ mice whether targeting toxic DM1 CUG repeats via a blocking mechanism for Mbnl1 displacement [35] or targeting a miR-23b binding site on the Mbnl1 3′UTR increasing Mbln1 protein levels using ASOs called blockmiRs [92]. In the case of PPMOs targeting the repetitive sequence, a single tail vein injection of Pip6a-PMO at 12.5 mg/kg induced a significant correction of Mbnl1-dependent splicing defects 2 weeks after treatment, with a 50% reduction in the number of RNA foci and a 60% decrease in CUGexp-RNA [35]. This is in contrast to three injections at 200 mg/kg of naked PMO which did not have any effect in the molecular phenotype. Repeated systemic injections of Pip6a-PMO at 12.5 mg/kg led to the complete correction of Cncl1 and Mbnl1 DM1 alternative splicing while heatmap results demonstrate a global gene expression correction in treated mice [35]. Moreover, hind-limb myotonia was completely reversed after treatment and the quantification of corrected splicing changes revealed long-lasting activity of Pip6a-PMO 26 weeks after treatment [35]. The Wood laboratory continued to develop cell-penetrating peptides with a wider therapeutic window than Pip6a, forming the basis of an MRC-University of Oxford spin-off company (PepGen) to take the peptide PMO chemistry to clinical trials. PepGen has recently started a phase 1 clinical trial of a peptide-conjugated oligonucleotide (PGN-EDO51) for the treatment of DMD and is intending to start a clinical trial with PGN-EDODM1 in DM1 patients [36].

## 4. Alternative Routes of Delivery

While advances in ASO chemical modification and conjugation have rendered the delivery of DM1 ASOs to skeletal and cardiac muscle more effective, delivering enough compound to the CNS to induce a clinically relevant effect remains a challenge. The majority of treatments in development for neuromuscular diseases are unable to cross the BBB upon systemic administration. Therefore, in order to circumvent this biological barrier, routes of administration that deliver ASOs directly into the CNS have been utilised for a number of diseases such as SMA [93], Parkinson’s disease [94] and Huntington disease (HD) [95].

The most advanced CNS treatments with ASOs in patients have been deployed via intrathecal administration, with Nusinersen, a 2′-MOE ASO for SMA, being approved by the FDA in 2016 [96]. More recently, in 2019, Tabrizi et al. [95] tested the 2′-MOE RG6042, also known as HTT_RX_, designed to reduce concentrations of Huntingtin (HTT) messenger RNA in HD [95]. Data from the phase 1/2a clinical trial (NCT02519036) in 46 adult patients with early HD showed an ASO-mediated HTT reduction of up to 30% 24 h after intrathecal administration. The compound demonstrated a good safety profile, with no evidence of adverse events after a dosing regimen of four repeated monthly intrathecal administrations; however, the phase 3 clinical trial (NCT03761849) was halted based on the benefit–risk profile.

Intracerebroventricular (ICV) administration has been attempted since the first stages of ASO development [97,98,99]. ICV delivery of ASOs is now being explored for DM1 in the DMSXL mouse model, a DM1 transgenic mouse model harbouring a human DMPK gene with over 1000 CTG repeats and displaying a CNS phenotype [28]. In the case of IONIS 486178 (a 16-nucleotide ASO gapmer containing BNA (cEt) modifications) [100], a single 75 µg ICV bolus administration of the ASO-induced *DMPK* mRNA knockdown, nuclear redistribution of MBNL1/2 and a reduction in RNA foci in the CNS of the DMSXL mouse model for at least 3 weeks [28]. Additionally, there was a correction in the phenotypic behavioural abnormalities. IONIS 486178 ASO showed distribution throughout the brain, with the greatest compound accumulations in the hypothalamus, cortex and cerebellum. Furthermore, no CNS inflammation or peripheral toxicity was detected post treatment, suggesting that the compound is well tolerated in adult mice [28,100]. These results are particularly promising for congenital or childhood DM1 patients that typically experience more severe CNS symptoms [101,102].

## 5. Future Challenges and Concluding Remarks

Nowadays, with the efficacy of antisense therapy being repeatedly demonstrated in DM1 models, the largest challenge remaining for this class of compounds is delivery to critically affected tissues in humans. Advancements in chemical modifications and bioconjugations have rendered overcoming this barrier more achievable, although safety and tolerability studies have shown that approaches to improve delivery and potency need to be carefully balanced with maintaining low toxicity. The challenge of delivery to the CNS at clinically relevant levels also remains and direct CNS administration routes may be necessary to ameliorate some of the symptoms experienced by patients. Nevertheless, with several ASO therapies currently in clinical trials, the outlook for achieving a clinically approved treatment for patients has never looked more promising.

## Data Availability

Data sharing is not applicable to this article.
